# Role of 18F-FDG-PET/CT in Combination With Neutrophil–Lymphocyte Ratio in the Diagnosis of Upper Urinary Tract Lesion: Can We Accurately Predict Malignant Tumor?

**DOI:** 10.3389/fonc.2021.615881

**Published:** 2021-09-22

**Authors:** Zhi-Bin Ke, Xiao-Dan Lin, Ye-Hui Chen, Yun-Zhi Lin, Shao-Hao Chen, Shao-Ming Chen, Yu Chen, Yong Wei, Qing-Shui Zheng, Xue-Yi Xue, Xiao-Dong Li, Ning Xu

**Affiliations:** ^1^Department of Urology, Urology Research Institute, The First Affiliated Hospital, Fujian Medical University, Fuzhou, China; ^2^Department of Nuclear Medicine, The First Affiliated Hospital of Fujian Medical University, Fuzhou, China; ^3^Cancer Bio-Immunotherapy Center, Fujian Medical University Cancer Hospital and Fujian Cancer Hospital, Fuzhou, China; ^4^Department of Medical Oncology, Fujian Medical University Cancer Hospital and Fujian Cancer Hospital, Fuzhou, China

**Keywords:** upper urinary tract lesions, neutrophil–lymphocyte ratio, 18F-FDG-PET/CT, diagnosis, malignant tumor

## Abstract

**Objective:**

To explore whether preoperative 18Fluorine-Fluorodeoxyglucose-positron emission tomography/computed tomography (18F-FDG-PET/CT) in combination with neutrophil–lymphocyte ratio (NLR) could accurately predict malignant lesions of upper urinary tract (UUT).

**Methods and Materials:**

The clinicopathologic data of a total of 252 patients with UUT lesions receiving surgical treatment at our center from January 2012 to November 2019 were retrospectively analyzed. All patients performed routine preoperative hematological examination, urine cytology, computed tomography urography (CTU), and 18F-FDG-PET/CT. Clinicopathologic data between 179 cases with malignancy (Group 1) and 73 cases with benign lesions (Group 2) were compared. Multivariate logistic regression analysis was used to explore the independent predictors of malignant UUT lesions. Receiver operating characteristic (ROC) curve was used to evaluate the predictive ability.

**Results:**

Among all patients, univariate analysis showed that NLR, hydronephrosis, CTU indicating malignancy, and PET/CT indicating malignancy were significantly associated with malignant UUT lesions; multivariate analysis revealed that NLR, CTU indicating malignancy, and PET/CT indicating malignancy were independent predictors of malignant UUT lesions; the area under ROC curve (AUC) of NLR, CTU, PET/CT, combining CTU and NLR, combining PET/CT and NLR, and combining PET/CT and CTU were 0.735, 0.788, 0.857, 0.863, 0.913, and 0.919, respectively, for postoperative pathological malignancy. Among 68 patients undergoing ureteroscopy biopsy, univariate analysis suggested that NLR, positive urine exfoliation cytology, CTU indicating malignancy, and PET/CT indicating malignancy were significantly associated with malignant UUT lesions; multivariate analysis demonstrated that positive urine cytology, PET/CT indicating malignancy, and NLR were independent predictors of malignant UUT lesions; the AUC of NLR, ureteroscopy biopsy, and combining PET/CT and NLR were 0.768, 0.853, and 0.839, respectively, for postoperative pathological malignancy.

**Conclusions:**

Combining preoperative NLR and PET/CT performed well in differentiating benign from malignant UUT lesions, which could not be identified by traditional imaging or urine cytology. Combining preoperative NLR and PET/CT could be used to reduce unnecessary ureteroscopy biopsy, which might result in tumor cell dissemination and risk of associated complications.

## Introduction

Upper urinary urothelial carcinoma (UTUC) accounts for 5%–10% of all urothelial cancer with high recurrence and progression rates (1–5). Radical nephroureterectomy (RNU) remains the standard treatment for UTUC (6, 7). Nephron-sparing surgery (NSS) and endoscopic treatment were regarded as an alternative therapy for strictly selected patients (7). However, the heterogeneity of UTUC in diagnosis and prognosis poses great challenges for clinical decision-making (4, 8). Hence, for the sake of early diagnosis and treatment of UTUC, it is of great importance to accurately distinguish benign from malignant upper urinary tract (UUT) lesions (3, 8).

There were numerous methods for the diagnosis of UTUC including urine cytology, magnetic resonance urography (MRU), computed tomography urography (CTU), and ureteroscopy. The specificity of urine cytology was reported to be 99%, but the sensitivity depends on the grade and stage of UTUC with a value ranging merely from 38% to 51% (9). The European Association of Urology (EAU) guidelines suggested that CTU is the preferred imaging method for diagnosing UTUC with higher sensitivity and specificity than MRU (7). However, the diameter of the lesion significantly affects the sensitivity of CTU. There were approximately 89% of lesions with a diameter of 3–5 mm that could be detected using CTU; however, only 40% of lesions < 3 mm in diameter could be found by CTU (9). Ureteroscopy (URS) allows ureteral masses biopsy under direct vision with higher specificity and sensitivity than CTU (10–13). However, ureteroscopy is an invasive procedure with a risk of multiple complications, including ureteral perforation, false tract formation, intraoperative and postoperative bleeding, and urinary tract infections (UTIs) (14, 15). Furthermore, diagnostic ureteroscopy would increase the risk of intravesical recurrence (3). Therefore, there is a lack of convenient methods for accurate diagnosis of upper urothelial cancer currently.

Studies have shown that inflammation levels are closely related to tumor staging, grading, and tumor cell proliferative activity (16). Neutrophil–lymphocyte ratio (NLR) is a simple, convenient, repeatable, and inexpensive indicator of inflammation. Recent studies have revealed that high level of NLR could indicate malignant degree of tumor and is associated with recurrence (17), metastasis (18), and poor prognosis (18–21) of tumors. NLR could be used for determining RNU or NSS, the extent of lymph node dissection, perioperative systemic treatment, and follow-up planning for UTUC patients (21). In addition, it has been reported that 18Fluorine-Fluorodeoxyglucose-positron emission tomography/computed tomography (18F-FDG-PET/CT) is more sensitive to the diagnosis of bladder cancer and UTUC patients compared with traditional imaging (22).

This retrospective study aims to explore whether the combination of preoperative 18F-FDG-PET/CT and NLR could accurately predict malignant lesions of upper urinary tract (UUT).

## Materials and Methods

This study was approved by the Ethics Committee of the First Affiliated Hospital of Fujian Medical University and written informed consent was provided by each included patient. We retrospectively collected the clinicopathologic data of 303 patients undergoing surgical treatment of UUT lesions from January 2012 to November 2019 in the First Affiliated Hospital of Fujian Medical University. All patients performed routine preoperative hematological examination, urine cytology, CTU, and 18F-FDG-PET/CT. NLR calculation: the peripheral blood neutrophil count/the peripheral lymphocyte count (NLR) (19, 23, 24).

Inclusion criteria were as follows: (1) imaging indicating upper urinary tract lesions; (2) underwent CTU before treatment; (3) underwent 18F-FDG-PET/CT before treatment; and (4) underwent urinary shedding cytology three times before treatment. Patients who met all of the above criteria were included in the analysis.

Exclusion criteria were as follows: (1) UTI before treatment; (2) underwent ureteroscopy or ureteral stenting implantation or nephrostomy before 18F-FDG-PET/CT; (3) underlying disease affecting NLR levels including infectious diseases (bacterial/viral infections), diabetes, hypertension, immunological diseases, and other chronic diseases (25, 26); (4) medication history affecting NLR levels (21); (5) white blood cells count > 10,000/μl or <3,000/μl before treatment; and (6) incomplete clinical data. Patients who met any of the above criteria were excluded from the study. Eventually, we excluded 51 cases according to the above exclusion criteria.

A total of 252 patients were included in the final analysis, including 179 patients with UUT malignancies (Group 1) and 73 patients with benign UUT lesions (Group 2). Clinicopathologic data between Group 1 and Group 2, including general information, hematological tests, urine cytology, imaging, and pathological examinations, were compared. Hydronephrosis before treatment was classified according to the Society of Fetal Urology (SFU) grading system (27, 28). The final pathological diagnosis was confirmed by surgical specimens. The TNM staging system formulated by the American Joint Committee on Cancer-Union International contre le Cancer was used for tumor staging (6).

Patients whose CTU images are inconsistent with urine cytology in determining tumor nature required further ureteroscopy biopsy, including the following three types: (1) CTU indicating benign lesions but urine cytology suggesting malignant lesions for at least one time; (2) CTU indicating malignant or uncertain lesions but consecutive urine cytology suggesting negative results for three times; and (3) CTU indicating uncertain results but urine cytology suggesting negative results for less than three times. Finally, a total of 68 patients (27%) required ureteroscopy biopsy.

### Examination Procedure of 18F-FDG-PET/CT

All patients fasted for at least 6 h and blood glucose levels were confirmed to be 80–120 mg/dl before intravenous injection of 18F-FDG. The GE Discovery ST 16 PET/CT scanner (General Electric Healthcare, Milwaukee, Wisconsin) was used. Radiochemical purity of 18F-FDG is greater than 95%. Approximately 500 ml of water is used to promote the excretion of FDG. We obtained the whole-body image precisely 90 min after administering 222–370 MBq (6–10 mCi) of 18F-FDG intravenously. Besides, PET imaging was amended for measured attention and reconstructed utilizing an ordered-subset expectation maximization iterative algorithm as suggested by the manufacturer. PET and CT images were then transferred to Siemens Syngo workstation for image registration fusion. The results were comprehensively analyzed based on the 18F-FDG metabolic distribution, morphological characteristics, and standardized uptake value in lesions and surrounding normal tissues. Two nuclear medicine physicians independently interpreted the imaging of 18F-FDG-PET/CT with a diagnostic consistency of 97.0%. Disagreement was resolved by the consultation of these two nuclear medicine physicians (6, 29).

### Statistical Method

Statistical analysis was performed using SPSS 26.0 software (IBM Corp., Armonk, NY, USA). Numerical data were analyzed using the chi-square test or Fisher’s test. Quantitative data that are not subject to the normal distribution were analyzed using the Mann–Whitney *U* test. Multivariate logistic regression analysis was used to determine the independent predictors for malignant UUT lesions. Receiver operating characteristic (ROC) curve was used to evaluate the diagnostic value of NLR in malignant UUT lesions. The Youden index was used to determine the optimal threshold of NLR in the diagnosis of malignant UUT lesions. ROC analysis was performed using MedCalc 18.2.1 software. *p* < 0.05 was considered statistically significant.

## Results

**Figure 1:**
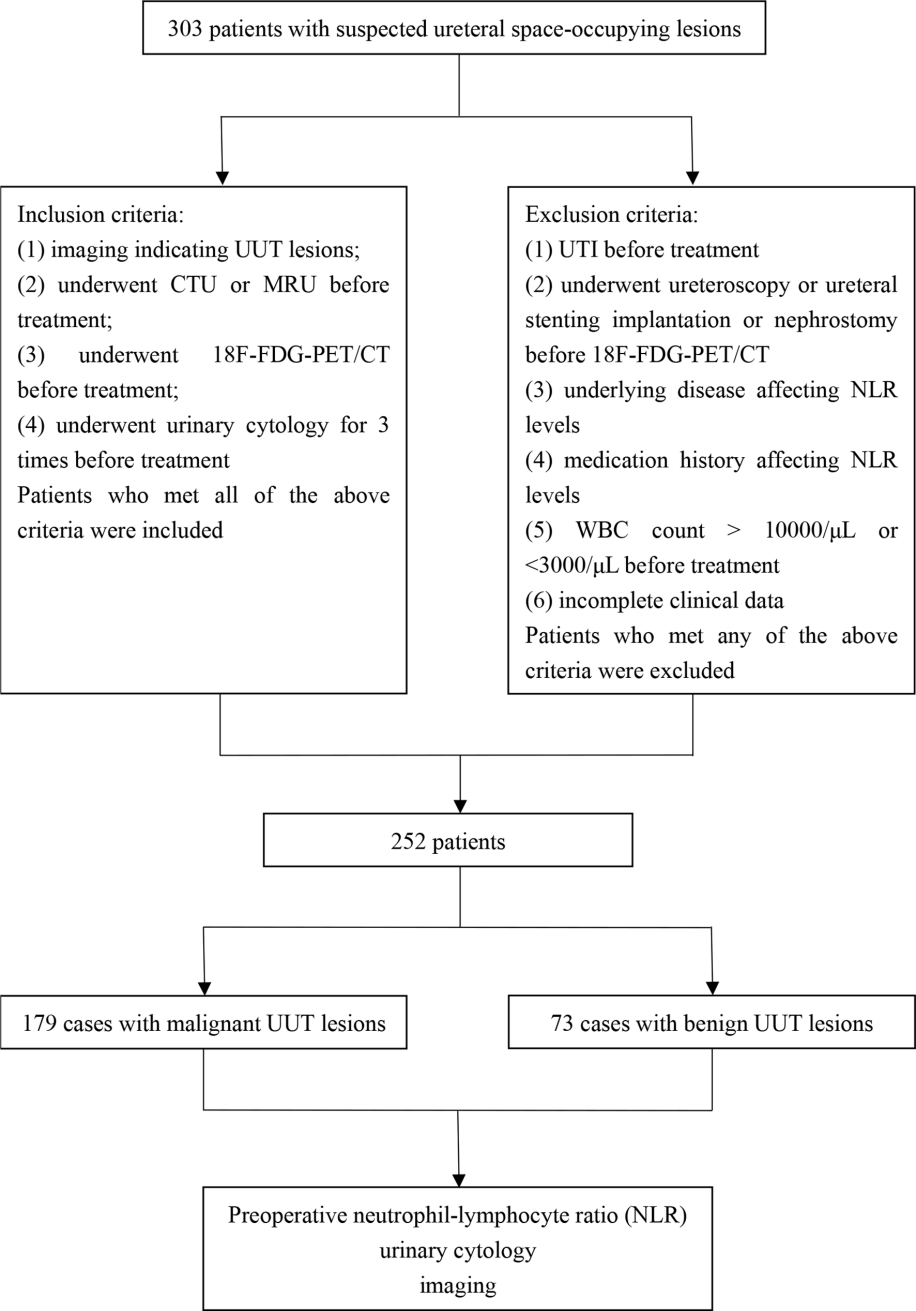
The flow chart of this study.

**Figure 2:**
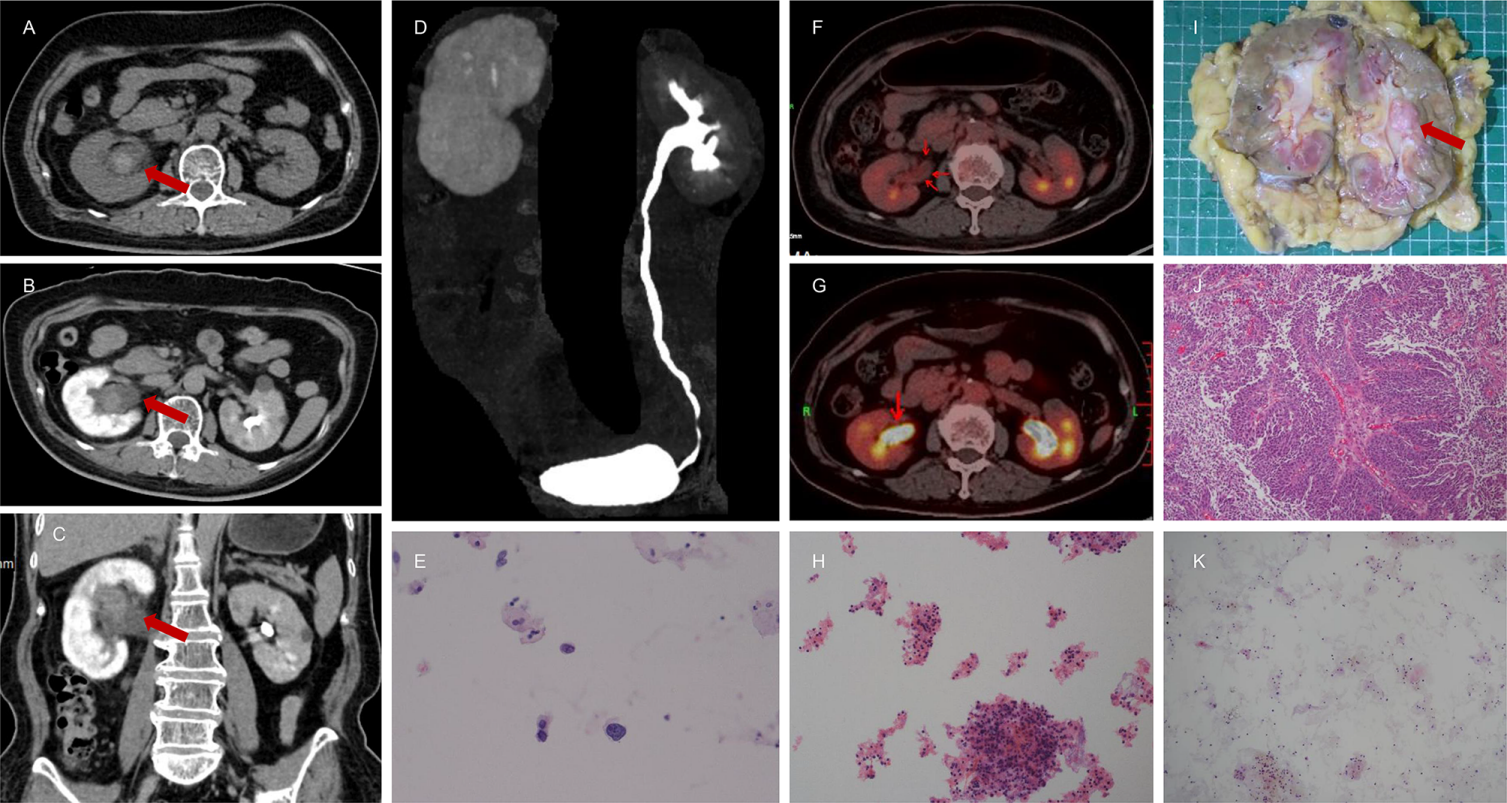
A 67-year-old female presented with intermittent painless gross hematuria for 3 months. The results of CTU indicated hematomas or neoplastic lesion (1.9 cm * 1.4 cm * 2.9 cm) in the right renal pelvis due to clear boundaries, slightly high density, slightly homogeneous enhancement, the inconspicuous filling defect, and obviously dilated upper urinary tract **(A–D)**. Magnetic resonance imaging (MRI) also suggested benign lesions in the right renal pelvis. Nuclear heterogeneous cells were not found for one time **(K)** but there were few suspicious nuclear heterogeneous cells for two times **(E, H)** in the histopathological analysis of urine cytology. However, the NLR value was 3.45. Therefore, this patient was referred to the Department of Nuclear Medicine for 18F-FDG PET/CT. It demonstrated intense 18F-FDG uptake in right renal pelvis **(F, G)**. Combining NLR and 18F-FDG PET/CT, this patient was highly suspected to have a malignant tumor of renal pelvis and subjected to right RNU. The final pathological report confirmed a diagnosis of right renal pelvic carcinoma **(I, J)**.

**Figure 3:**
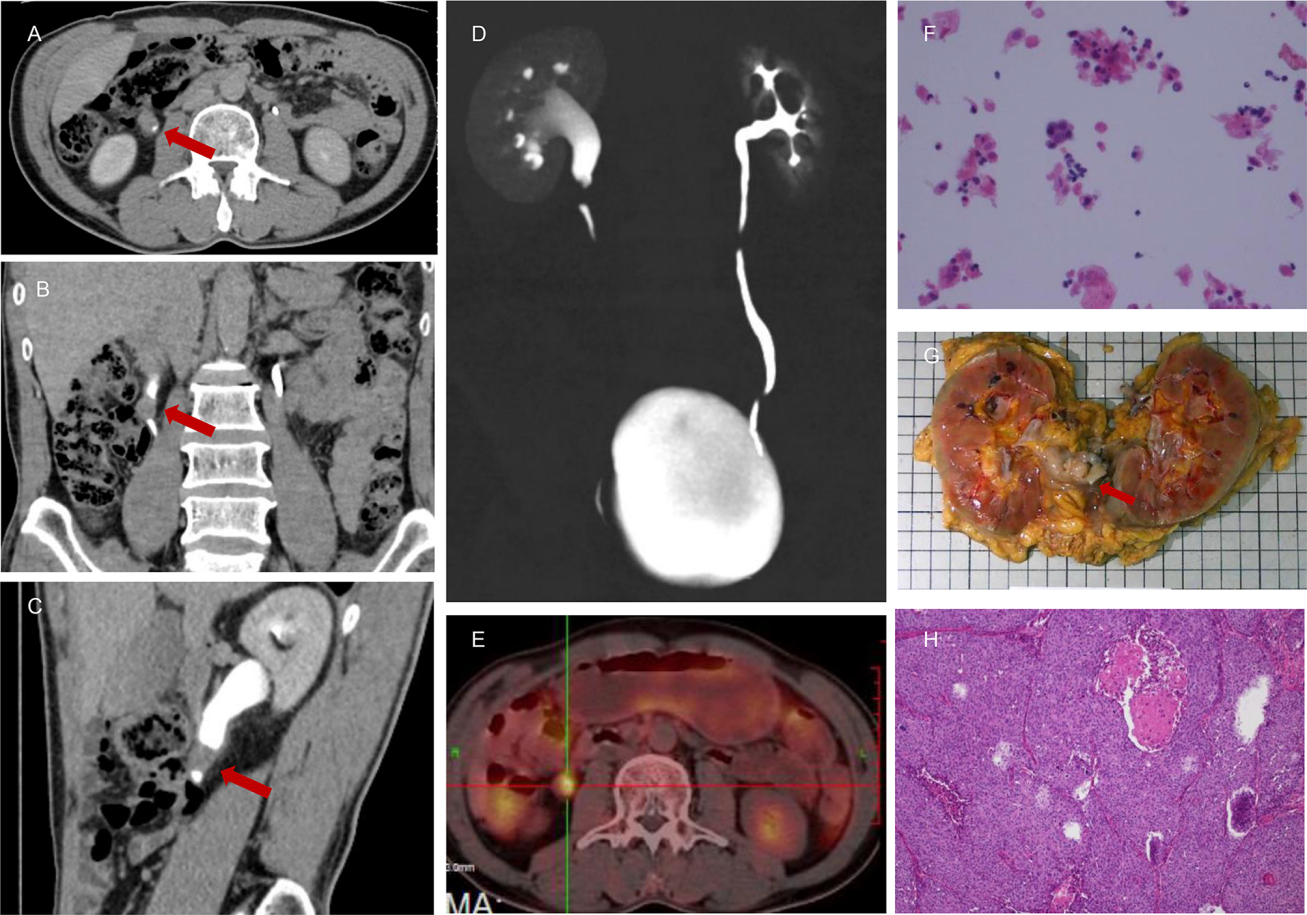
A 55-year-old male presented with persistent painless gross hematuria for 1 month. The results of CTU indicated malignant tumor (1.2 cm * 1.7 cm) of the right ureter **(A–D)**. There were nuclear heterogeneous cells suggested by urine cytology that were regarded as malignant tumor cells **(F)**, and the NLR value was 3.99. Intense 18F-FDG uptake was also noted in the right upper ureter **(E)**, which indicated a high probability of malignant tumor. This patient was subjected to right RNU, and the final pathological report confirmed a diagnosis of right upper ureteral carcinoma **(G, H)**.

The flow chart of this study is shown in **Figure 1**. Finally, a total of 252 patients were included in this study, namely, 179 cases of UUT malignancies (Group 1) and 73 cases of benign UUT lesions (Group 2). The postoperative pathological data and surgical procedures are presented in **Table 1**. The baseline clinicopathologic data of these two groups are shown in **Table 2**. The imaging and pathological data of two typical cases are shown in **Figures 2** and **3**.

**Table 1:** Comparison of postoperative pathological data and surgical options.

	Group 1 (benign, 73 cases)		Group 2 (malignant, 179 cases)	
Postoperative pathological data (*n*)	Polyps	33	Urothelial carcinoma	161
	Papilloma	15	Squamous cell carcinoma	7
	Liomyoma	10	Adenocarcinoma	3
	Fibroma	6	Sarcoma	3
	Inflammatory polyps	3	Metastatic malignancy	2
	Other benign tumors	6	Other malignant tumors	3
Surgical procedure (*n*)	Ureteroscopic holmium laser resection	44	Ureteroscopic holmium laser resection	6
	Partial ureterectomy	27	Partial ureterectomy	15
	Radical nephroureterectomy	2	Radical nephroureterectomy	158

**Table 2:** Clinicopathologic data between the benign group and the malignant group of UUT lesions.

	Overall cohort		*p*	Patients requiring ureteroscopy biopsy		*p*
	Benign (73)	Malignant (179)		Benign (35)	Malignant (33)	
Age (years)	40.01 ± 11.85	45.73 ± 11.14	0.277	41.89 ± 9.60	46.15 ± 10.25	0.081
Gender (*n*)						
Male	36	79	0.454	17	13	0.446
Female	37	100		18	20	
Tumor side (*n*)						
Left	35	87	0.924	17	22	0.132
Right	38	92		18	11	
Tumor location (*n*)						
Pelvis	24	52	0.174	12	10	0.891
Ureter	35	106		16	17	
Both	14	21		7	6	
T stage (*n*)						
pTis	–	12	NA	–	2	NA
pTa	–	23		–	2	
pT1	–	42		–	10	
pT2	–	45		–	9	
pT3	–	37		–	4	
pT4	–	20		–	6	
N stage (*n*)						
pN0	–	132	NA	–	22	NA
pNx	–	47		–	11	
NLR						
>2.5	24	143	<0.001*	12	29	<0.001*
≤2.5	49	36		23	4	
Cytology (*n*)						
Positive	32	68	0.389	23	4	<0.001*
Negative	41	111		12	29	
Hydronephrosis (*n*)						
Grade 0–1	8	39	0.045*	0	5	0.118
Grade 2	35	56		17	13	
Grade 3	21	53		13	10	
Grade 4	9	31		5	5	
CTU (*n*)						
Malignant	13	135	<0.001*	7	19	<0.001*
Benign	53	12		23	4	
Uncertain	7	32		5	10	
PET/CT (*n*)						
Malignant	3	135	<0.001*	1	16	<0.001*
Benign	67	37		34	16	
Uncertain	3	7		0	1	
Diagnostic ureteroscope (*n*)						
Rigid	–	–	NA	21	22	0.569
Flexible	–	–		14	11	
Ureteroscopy biopsy (*n*)						
Malignant	–	–	NA	5	28	<0.001*
Benign	–	–		30	5	

*p < 0.05; NLR, neutrophil–lymphocyte ratio; UUT, upper urinary tract, CTU, computed tomography urography; PET/CT, positron emission tomography/computed tomography.

NA, Not Applicable.

**Figure 4:**
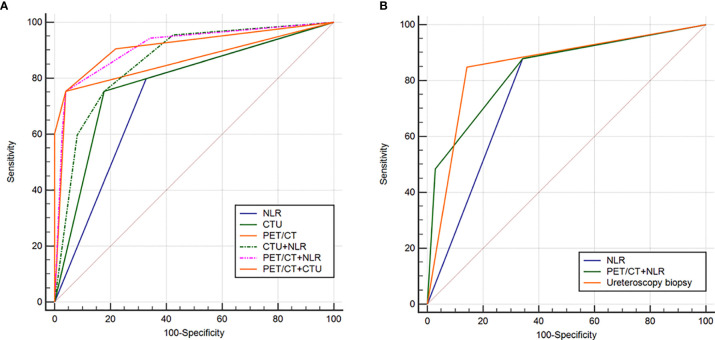
Receiver operating characteristic (ROC) curve analysis to evaluate the predictive ability of various diagnostic methods in distinguishing benign from malignant lesions of upper urinary tract in the whole cohort **(A)** and patients requiring ureteroscopy biopsy **(B)**.

Among all patients, univariate analysis showed that NLR, hydronephrosis, CTU indicating malignancy, and PET/CT indicating malignancy were associated with malignant UUT lesions (**Table 2**); multivariate logistic analysis revealed that NLR, CTU indicating malignancy, and PET/CT indicating malignancy were independent predictors of UUT malignancy (**Table 3**); the area under ROC curve (AUC) of NLR, CTU, PET/CT, combining CTU and NLR, combining PET/CT and NLR, and combining PET/CT and CTU were 0.735, 0.788, 0.857, 0.863, 0.913, and 0.919, respectively, for postoperative pathological malignancy (**Table 4** and **Figure 4A**).

**Table 3:** Multivariate logistic regression analysis exploring independent predictors of malignant UUT lesions.

	Overall patients (benign/malignant)			Patients requiring ureteroscopy biopsy (benign/malignant)		
	*p*	OR	95% CI	*p*	OR	95% CI
NLR group	<0.001*	5.785	2.398–13.954	0.011*	7.701	1.606–36.926
Positive urine cytology	–	–	–	0.008*	0.063	0.008–0.484
Hydronephrosis grade	0.621	0.889	0.559–1.416	–	–	–
CTU indicating malignancy	<0.001*	7.687	3.165–18.673	0.791	1.266	0.222–7.205
PET-CT indicating malignancy	<0.001*	42.575	11.729–154.534	0.028*	16.278	1.347–196.736

*p < 0.05; NLR, neutrophil–lymphocyte ratio; UUT, upper urinary tract; CTU, computed tomography urography; PET/CT, positron emission tomography/computed tomography.

**Table 4:** Evaluation of predictive power of various methods to diagnose malignant lesions of UUT using ROC curve.

	AUC (95%CI)	
	Overall	Patients requiring ureteroscopy biopsy
NLR	0.735 (0.676–0.788)	0.768 (0.650–0.862)
CTU	0.788 (0.732–0.837)	–
PET/CT	0.857 (0.807–0.897)	–
CTU+NLR	0.863 (0.814–0.903)	–
PET/CT+NLR	0.913 (0.871–0.945)	0.839 (0.729–0.917)
PET/CT+CTU	0.919 (0.879–0.950)	–
Ureteroscopy biopsy	–	0.853 (0.746–0.927)

NLR, neutrophil–lymphocyte ratio; UUT, upper urinary tract; ROC, receiver operating characteristic curve; AUC, the area under ROC curve; CTU, computed tomography urography; PET/CT, positron emission tomography/computed tomography.

Among 68 patients (27%) requiring ureteroscopy biopsy, univariate analysis showed that NLR, positive urine exfoliation cytology, CTU indicating malignancy, and PET/CT indicating malignancy were predictors of malignant UUT lesions (**Table 2**); multivariate analysis revealed that positive urine cytology, PET/CT suggesting malignancy, and NLR were independent predictors of malignant UUT lesions (**Table 3**). The AUC of NLR, ureteroscopy biopsy, and combining PET/CT and NLR were 0.768, 0.853, and 0.839, respectively, for postoperative pathological malignancy (**Table 4** and **Figure 4B**).

## Discussion

Ureteroscopy biopsy is one of the most important diagnostic methods for UUT lesions. However, the EAU guideline suggested ureteroscopy biopsy at merely C recommendation level (7). Ureteroscopy could lead to potential tumor progression and metastasis, increasing the risk of tumor recurrence after RNU (14, 15). During diagnostic ureteroscopy, flushing pressure under high-pressure saline solution infusion increases the risk of developing submucosal tumor spread, which can lead to microvascular or lymphatic metastasis (30). Besides, tumor cells could flow into the bladder accompanied with saline, which might implant into the mucosa and increase the risk of intravesical recurrence after RNU (3, 14, 30, 31). In addition, diagnostic ureteroscopy would delay the implementation of RNU, which might provide time for tumor spread due to ureteroscopy and lead to poor prognosis (30). In this study, we found that the AUC of ureteroscopy biopsy was similar with a combination of preoperative PET/CT and NLR, indicating that combining preoperative PET/CT and NLR was similar with ureteroscopy biopsy in diagnosing malignant lesions of UUT.

Identifying pathological properties by preoperative imaging would contribute to reduce unnecessary ureteroscopy biopsy, intraoperative ureteral exploration, and RNU, which might result in tumor cell dissemination (3). However, using traditional radiological methods to distinguish benign or malignant tumors occasionally lead to inappropriate surgical decisions (32). It is reported that about 2.9%–12.8% of suspected UUT masses were finally confirmed as benign lesions after RNU (3). In this study, among all patients, CTU indicating malignancy was an independent predictor of UUT malignancy. However, among patients requiring ureteroscopy biopsy, CTU indicating malignancy was not an independent risk factor for UUT malignant lesions.

NLR is defined as the ratio of neutrophil to lymphocyte count in peripheral blood (32). Compared with CTCs, NLR is economic, repeatable, and easy to measure, which could be widely used in clinical practice. NLR reflects the balance between neutropenia and lymphopenia caused by anti-tumor immunity. Granulocyte colony-stimulating factor (G-CSF) secreted by tumor cells contributed to the production of bone marrow neutrophils and recruitment of neutrophils into the tumor microenvironment. These tumor-associated neutrophils play a tumor-promoting role *via* a variety of ways, including extracellular matrix (ECM) remodeling, secreting tumor-promoting factors, enhancing cancer cell invasion, promoting angiogenesis and lymphangiogenesis, facilitating cancer cell proliferation, inhibiting lymphocytes activity, and suppression of anti-tumor immune surveillance (18, 21, 33). Besides, lymphocytes play an important role in cancer immune surveillance. Lymphopenia may lead to a poor immune response to tumors, which is conducive to tumor aggressiveness and progression. Thus, high NLR level indicates a decrease in lymphocyte-mediated anti-tumor immune responses and an increase in neutrophil-dependent inflammatory responses, which might lead to cancer invasiveness, progression, and poor prognosis (24, 32, 34, 35).

EAU guidelines recommend NLR as a predictor of cancer-specific survival (CSS) after UTUC treatment (7). Recent studies also reported that high levels of both preoperative and postoperative NLR were predictive of poor prognosis in patients with UTUC (8, 21, 24, 34–36). High levels of NLR before treatment were significantly associated with high grade (G3) and high stage (T3–4) of UTUC. This may be due to the biologically aggressive behavior of high-grade tumor that tends to stimulate the immune system and cause a stronger inflammatory response (37). In addition, UTUC could invade the entire ureteral wall and surrounding tissue easily (38). It was reported that there were about 50% of UTUC patients accompanied with muscular invasion and 30% of UTUC patients accompanied with lymph node involvement at the time of diagnosis (1). Therefore, we speculated that UTUC was easily activated by anti-tumor immunity and led to an increase in NLR compared with benign tumor due to high invasiveness. Previous studies have reported that NLR could serve as a predictor of presence of malignant tumors, local progression, and lymph node metastases (16, 34). This is the first study to explore whether NLR could be used to differentiate benign from malignant lesions of UUT. We found that preoperative NLR level was significantly higher in malignant lesions compared with benign lesions of UUT.

It was reported that malignant tumors were frequently presented as hypermetabolic lesions in 18F-FDG-PET/CT due to high invasiveness and growth activity. Therefore, 18F-FDG-PET/CT could be used to diagnose UTUC and predict the presence of muscularis invasion or lymph node metastasis (39, 40), which might help guide perioperative treatment decisions (for example, intraoperative lymph node dissection or chemotherapy) (6). Previous studies have reported that the combination of NLR with other tools could be used to help make clinical decision (35, 41). This is the first attempt to investigate whether a combination of NLR and 18F-FDG-PET/CT could be used to differentiate benign from malignant lesions of UUT. The results revealed that the AUC for PET-CT+CTU and PET-CT+NLR was increased compared with CTU+NLR or PET-CT alone or CTU alone or NLR alone. The result revealed that the predictive power of combining preoperative PET/CT and NLR was superior to CTU+NLR or PET-CT alone or CTU alone or NLR alone in diagnosing malignant lesions of UUT. Besides, the AUC for PET-CT+NLR was similar to PET-CT+CTU, indicating a similar predictive power. Therefore, considering the convenience and bargain price of NLR, the examination of CTU is unnecessary if patients have undergone PET-CT.

In this study, we found that preoperative hydronephrosis was not a predictor of malignant tumors of UUT. The possible reason is that the patients included in this study were mostly at early stage. At the early stages of tumor development, there was no significant difference in the impact on UUT obstruction between benign and malignant lesions. However, preoperative hydronephrosis frequently indicates a high probability of muscle infiltration of UTUC for muscle layer invasion could cause more severe intraluminal obstruction (6, 42, 43). This study demonstrated that the combination of NLR and 18F-FDG-PET/CT was an independent predictor of malignant lesions of UUT. Therefore, the combination of NLR and PET/CT might be superior to hydronephrosis in differentiating benign and malignant UUT masses.

There were several limitations in this study. Firstly, this is a single-center retrospective study with limited sample size using non-randomized methods. External validation of our results using randomized prospective methods with a larger sample size is needed to evaluate further in the future. Secondly, the cutoff value of NLR before treatment remains uncertain (41). Vartolomei et al. found that numerous studies related to UTUC defined the cutoff NLR value at least ≥ 2.2 (21). It is also reported that most studies defined cutoff NLR value as ≥2.5 (19, 23, 24). Thirdly, there were numerous risk factors affecting NLR levels including diabetes, hypertension, smoking, stress reaction, and other underlying infectious diseases (25, 26, 32). However, we could only reduce the impact on research error through detailed previous history and personal history investigation. Fourthly, some occult UUT infection patients with normal white blood cell count and without fever might also be included in this study, which would affect the results. However, highly aggressive malignancies are more likely to develop hydronephrosis by blocking UUT. The obstruction of UUT would lead to occult infection and inflammatory reaction. This is the potential mechanism of upper UTI. This also revealed that malignant ureteral tumors are more likely to provoke body inflammatory reaction, either anti-tumor immunity or UUT inflammation, and infection triggered by tumor compression (37).

## Conclusions

Combining preoperative PET/CT and NLR performed well in differentiating benign from malignant UUT lesions that could not be identified by traditional imaging or urine cytology. Combining preoperative PET/CT and NLR could be used to reduce unnecessary ureteroscopy biopsy, which might result in tumor cell dissemination.

## Data Availability Statement

The original contributions presented in the study are included in the article/supplementary material. Further inquiries can be directed to the corresponding authors.

## Ethics Statement

This study was approved by the Ethics Committee of the First Affiliated Hospital of Fujian Medical University. The patients/participants provided their written informed consent to participate in this study.

## Author Contributions

All authors listed have made a substantial, direct, and intellectual contribution to the work and approved it for publication.

## Funding

This study was supported by the Special Funding Project of Fujian Medical University (Grant number: 2019B030).

## Conflict of Interest

The authors declare that the research was conducted in the absence of any commercial or financial relationships that could be construed as a potential conflict of interest.

## Publisher’s Note

All claims expressed in this article are solely those of the authors and do not necessarily represent those of their affiliated organizations, or those of the publisher, the editors and the reviewers. Any product that may be evaluated in this article, or claim that may be made by its manufacturer, is not guaranteed or endorsed by the publisher.
